# Oncolytic adenovirus mediated Survivin knockdown by RNA interference suppresses human colorectal carcinoma growth in vitro and in vivo

**DOI:** 10.1186/1756-9966-28-81

**Published:** 2009-06-15

**Authors:** Wei Shen, Chun-Yi Wang, Xue-Hu Wang, Zhong-Xue Fu

**Affiliations:** 1 Department of General Surgery, The First Affiliated Hospital, Chongqing Medical University, Chongqing 400016, PR China; 2 Key laboratory of general surgery, The First Affiliated Hospital, Chongqing Medical University, Chongqing 400016, PR China

## Abstract

**Background:**

Colorectal cancer is a one of the most common alimentary malignancies. Survivin has been proved by many studies to be an ideal target for cancer gene therapy because of its strong anti-apoptotic effect. The reduction of Survivin expression by means of chemically synthesized small interfering RNA or small hairpin RNA expressed from plasmid and resulted growth inhibition of cancer cells had been proved by many studies including ours, but the transfection efficiency was not encouraging. So for the first time we constructed the Survivin shRNA into an oncolytic adenovirus, tested its effects on colorectal cancer cell lines and nude mice xenograft model.

**Methods:**

In this study, we constructed an oncolytic adenovirus with a Survivin targeted small hairpin RNA and a reporter gene (ZD55-Sur-EGFP). The expression of Survivin mRNA and protein were analyzed by RT-PCR and western blot. The cell growth and apoptosis were tested by in vitro cytopathic assay, MTT assay and flow cytometry respectively. The effect of the constructed virus on xenograft model was evaluated by tumor volume and western blot analysis.

**Results:**

ZD55-Sur-EGFP replicated in cancer cells specifically, reduced the expression of Survivin mRNA and protein expression effectively (P < 0.0001), induced cancer cell apoptosis and inhibited SW480 cell growth both in vitro and in vivo significantly.

**Conclusion:**

We conclude Survivin RNA interference combining with oncolytic adenovirus virotherapy to be a promising treatment for colorectal cancer.

## Background

Colorectal cancer (CRC) is the second leading cause of cancer-related deaths in the US and the incidence is increasing rather rapidly in developing countries including China [1]. Traditional treatments for colorectal cancer such as surgical resection and chemotherapy do not increase the survival rate satisfactory enough. There are still 50% patients died from tumor recurrence and metastasis. It is of great importance to find a new therapeutics against colorectal cancer.

Survivin, a member of the inhibitor of apoptosis protein (IAP) family, is expressed highly in most human tumors and fetal tissues, but is barely detectable in terminally differentiated cells [2]. The Survivin protein functions to inhibit caspase activation by interacting with caspases via baculovirus IAP repeat domains, therefore leading to negative regulation of apoptosis [3]. There was evidence by cDNA microarray that Survivin plays an important role in pathogenesis of colorectal cancer [4]. Several reports had successfully inhibited cancer cell growth by applying Survivin antagonists, antisense oligonuceotides or Survivin RNA interferences [5-7]. Thus Survivin is considered as an ideal target for colorectal cancer gene therapy [8].

ONYX-015, a well known E1B-55 kDa deleted adenovirus, has been used in clinical trials and achieved encouraging results. However, the therapeutic efficacy of ONYX-015 is limited when it is used as a single agent [9,10]. So we constructed a new E1B-55 kDa deleted adenovirus with a cloning site for exogenous gene, which offered a possibility for treatment of carcinomas with both oncolytic adenovirus and specific gene targeted RNA interference. We showed that the construct, ZD55-Sur-EGFP, specifically replicated in colorectal cancer cells, induced apoptosis and attenuated cancer cell growth both in vitro and in nude mice. ZD55-Sur-EGFP may be a promising therapy for colorectal cancer.

## Methods

### Construction of Survivin shRNA expression plasmid

A pair of short hairpin RNA (shRNA) targeting Survivin [GeneBank accession NM_001168] which had been reported [6] was constructed. The sequence was a 19 nt small interfering RNA: GGCTGGCTTCATCCACTGC (86–104) with a ring sequence of 9 base pairs connecting the sense and antisense strands (TTCAAGAGA). The shRNA was constructed into pMD-18T plasmid (TaKaRa), namely pMD-18T-S. The sequence was not homologous with any human coding gene by BLAST analysis.

### Cell lines and cell culture

Human colon adenocacinoma cell lines SW480, LoVo and intestinal epithelial cell (IEC) were obtained from Shanghai Cell Collection (Shanghai, China), HEK293 cells were purchased from Mircrobix Biosystems Ltd. (Canada). Cells were routinely cultured in Dulbecco's modified Eagle's media (Gibco) supplemented with 10% (vol/vol) fetal bovine serum (Gibco) at 37°C in a humidified incubator containing 5% CO_2_.

### Adenovirus construction

We constructed an E1b-55 kDa deleted oncolytic adenovirus construction plasmid pZD55 as reported [11] and it was reserved in our laboratory, but we added a reporter gene expressing enhanced green fluorescence protein (EGFP) which allowed for tittering and measuring of infection efficiency in transfected cells. Briefly, pIRES-EGFP (Clontech) was cut with EcoRI and XbaI to get the EGFP fragment. Then the EGFP segment was ligated into pCA13 (Microbix Biosystems) and pZD55 respectively to form pCA13-EGFP and pZD55-EGFP. After that, the Survivin shRNA expression cassette was excised from pMD-18T-Sur with XhoI and BamHI, first subcloned into pCA13-EGFP to form pCA13-Sur-EGFP. Then the expression cassette containing the Survivin shRNA controlled by the human CMV promoter and reporter gene EGFP were cut with Bgl II and subcloned into pZD55 to construct pZD55-Sur-EGFP. Oncolytic adenoviruses ZD55-Sur-EGFP, ZD55-EGFP, replication deficiency adenovirus AD-Sur-EGFP, AD-EGFP were generated by homologous recombination between pZD55-Sur-EGFP, pZD55-EGFP, pCA13-Sur-EGFP, pCA13-EGFP and the adenovirus packaging plasmid pBHGE3 (Microbix Biosystems) respectively. Viruses were purified by ultracentrifugation with cesium chloride. The titers were determined by cytopathic effect (CPE) on HEK293 cells in a 96-well plate by a fluorescence microscope.

### Detection of adenoviruses in cells

SW480 and LoVo cells as well as intestinal epithelial cells (IEC) were plated at 10^5 ^cells per 6 cm dish and infected with ZD55-Sur-EGFP or AD-Sur-EGFP for 48 h and 72 h. The expression of enhanced green fluorescent protein (EGFP) was accessed by a Zeiss fluorescence microscope coupled with a digital camera photo apparatus.

### RT-PCR analysis

Total RNA from transfected cells was isolated using TRIzol (Invitrogen) as recommended by the manufacturer. RT-PCR was used for the analysis of Survivin mRNA with GAPDH as an internal control. Primers for Survivin were as follow: forward primer 5'-GAC CAC CGC ATC TCT ACA TTC-3', reverse primer 5'-GTT CTT GGC TCT TTC TCT GTCC-3'. The GAPDH primers were forward 5'-ACC ACA GTC CAT GCC ATC AC-3' and reverse 5'-TCC ACC ACC CTG TTG CTG TA-3'. Reactions were performed in accordance with the standard protocol. PCR was performed by initial denaturation at 94°C for 5 min followed by 35 cycles of 30 s at 94°C, 30 s at 58°C and 60 s at 72°C. The products were separated by electrophoresis in 2% agarose and visualized with ethidium bromide. Experiments were performed in triplicate.

### Western blot analysis

Cells were transfected with adenoviruses and incubated for 48 h. After that they were harvested and the protein extracts were separated via sodium dodecyl sulfate-polyacdene gel electrophoresis and transferred onto nitrocellulose membranes. The membranes were then blocked with rabbit anti-Survivin, Ad2 E1A, β-actin (Santa Cruz), XIAP (Sigma) and caspase-3 (Beyotime, China) primary polyclonal antibodies respectively at 4°C overnight. After washing with PBS containing 0.05% Tween 20 the membranes were incubated with secondary antibody (goat anti-rabbit, Santa Cruz) for 2 h. They were visualized by chemiluminescence system according to manufacturer's instruction.

### In vitro cytopathic assay

Cells were grown subconfluently and infected with adenoviruses with indicated MOIs. 5 days later, the medium was removed and the cells were washed with PBS twice, exposed to Coomassie brilliant blue and then washed with distilled water. The result was documented as photographs.

### MTT cell viability assay

To quantify the cytopathic effect, MTT assay was performed. Cells were seeded in 96-well plates for 24 h at 1 × 10^4 ^per well. After 1 to 5 days of various viruses infection, 15 μl MTT (5 mg/ml in PBS) was added to each well for 4 h incubation at 37°C followed by the addition of 150 μl DMSO. Absorbance at 570 nm was measured for cell viability in each well.

### Flow cytometry evaluation

Apoptosis of cells infected with adenoviruses at MOI of 5 was determined by flow cytometry (FCM) using Annexin V: PE Apoptosis Detection Kit I (BD Biosciences, USA) according to manufacturer's instruction. Briefly, Cells were washed twice with cold PBS and resuspended in binding buffer at a concentration of 1 × 10^5 ^cells/ml. Then they were incubated with 5 μl of PE Annexin V and 5 μl of 7-AAD for 15 min at room temperature in the dark. At last, 400 μl of binding buffer was added and cells were analyzed by flow cytometry.

### Animal studies

Five-week-old, female BALBC/C nude mice were obtained from the Laboratory Animal Center of Chongqing Medical University. They were maintained in the specific pathogen free unit under isothermal conditions. All experimental procedures were carried out in accordance with the National Institute of Health Guide for the Care and Use of Laboratory Animals.

5 × 10^6 ^SW480 cells suspended in 0.1 ml serum free medium were implanted subcutaneously into the flank of nude mice. When tumors size reached about 100 mm^3^, mice were randomly divided into 5 groups with 6 mice in each group. ZD55-Sur-EGFP, ZD55-EGFP, AD-Sur-EGFP and AD-EGFP were injected through the tail vein with 5 × 10^8 ^PFU adenoviruses suspended in 100 μl PBS or 100 μl PBS alone for 3 days. Tumors were monitored by measuring tumor volume with a caliper. The volume was calculated by the formula: V (mm^3^) = length × width^2^/2. After 60 days experiment, the tumors were harvested for western blot analysis.

### Survivin protein expression in xenograft tumor

Snap-frozen tumor samples were homogenized mechanically in a buffer (150 mM sodium chloride, 0.1 M Tris (pH 8), 1% Tween-20, 50 mM diethyldithiocarbamic acid, 1 mM EDTA pH 8) containing protease inhibitors, before sonication and centrifugation at 4°C for 3 min. The following steps were the same as above mentioned in the western blot analysis part.

### Statistical analysis

All data were displayed as Mean ± S0D, analyzed via analysis of variance and Student t test, and processed by the statistical software SPSS 13.0. Statistical significance was assumed when p < 0.05.

## Results

### Adenovirus construction and identification

**Figure 1:**
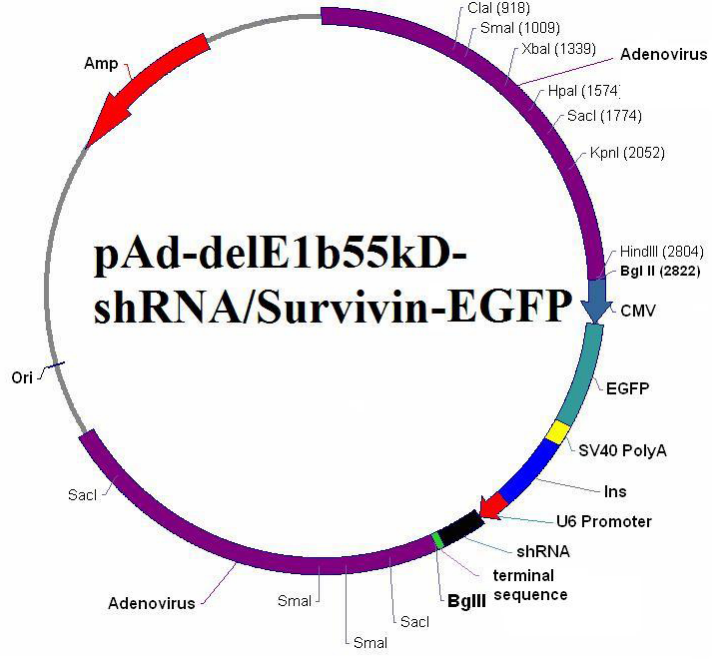
**The schematic presentation of ZD55-Sur-EGFP**. The E1B-55KD gene was replaced by Survivin-shRNA sequence expression cassette and EGFP.

**Figure 2:**
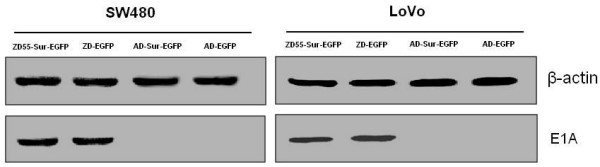
**E1A expression in SW480 and LoVo cells infected with ZD55-Sur-EGFP, ZD55-EGFP, AD-Sur-EGFP and AD-EGFP by immunoblot**. AD-Sur-EGFP and AD-EGFP were E1A deleted viruses, the E1A protein was absent in this analysis.

The recombinant adenoviral vector plasmid pZD55 had been constructed and reserved in our laboratory. Recombinant oncolytic adenovirus ZD55-Sur-EGFP was constructed by homologous recombination between pZD55-Sur-EGFP and the packaging plasmid pBHGE3. The schematic picture shows the recombinant ZD55-Sur-EGFP (Shown in Fig 1). The result was confirmed by restrictive enzyme digestion assay and sequence assay. E1A expression was also examined by immunoblot with SW480 and LoVo cells infected with various adenoviruses, shown in Fig 2. Results showed cells transfected with oncolytic viruses expressed E1A protein.

### Reporter gene assay in vitro

**Figure 3:**
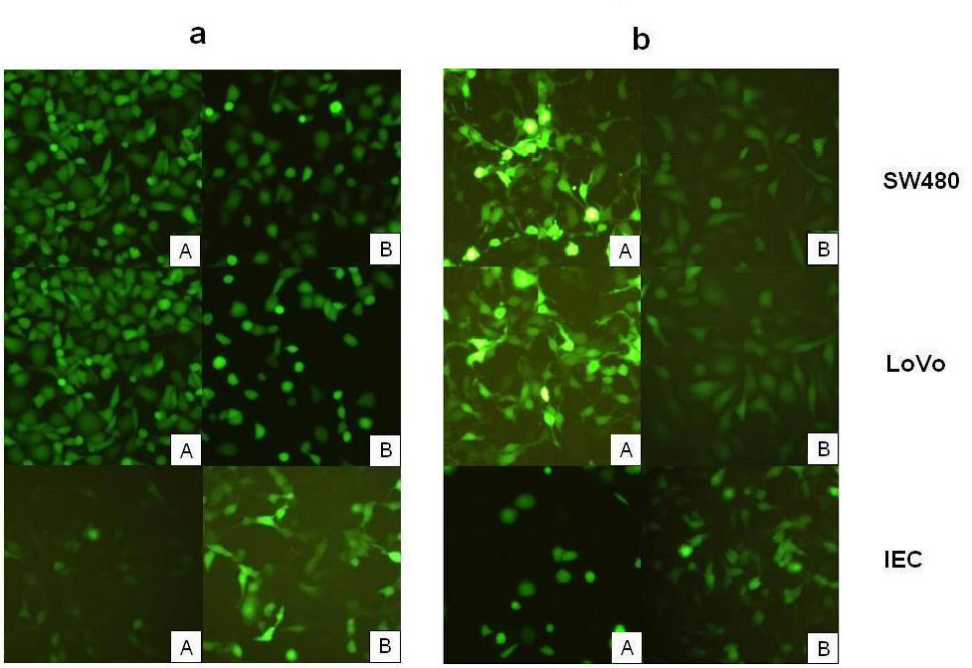
**SW480 and LoVo cells as well as IEC cells were plated at 10^5 ^cells per 6 cm dishes and infected with ZD55-Sur-EGFP (A) or AD-Sur-EGFP (B) for 48 h (a) or 72 h (b)**. Then the cells were observed through a fluorescence microscope. ZD55-Sur-EGFP showed much stronger affinity to SW480 cells than AD-Sur-EGFP, but it rarely replicated in normal cells IEC at 24 h post infection. After 72 h, the cells infected with ZD55-Sur-EGFP became lysed but there was little change in the morphology of AD-Sur-EGFP infected cells. (Original magnification ×200).

As shown in Fig 3a, the ZD55-Sur-EGFP demonstrated a high specificity to cancer cells. After 48 h, stronger green fluorescence was observed in SW480 and LoVo cells infected with ZD55-Sur-EGFP than with AD-Sur-EGFP at MOI of 5. On the contrary, ZD55-Sur-EGFP showed much lower affinity to normal IEC cells compared with AD-Sur-EGFP. After 72 h, the cancer cells infected with ZD55-Sur-EGFP became lysed but there was little change in the morphology of AD-Sur-EGFP infected cells.

### Inhibition of Survivin gene expression

**Figure 4:**
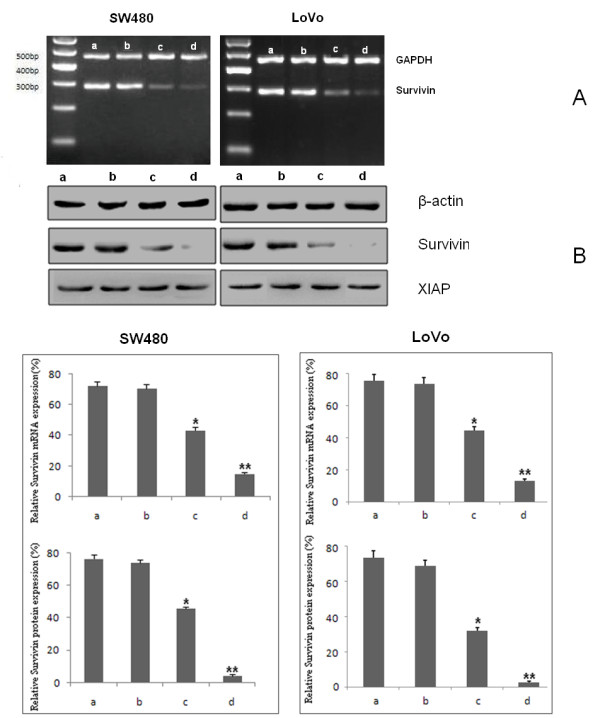
**Inhibition of Survivin mRNA and protein expression in SW480 and LoVo cells**. The cancer cells were treated with ZD55-Sur-EGFP, ZD55-EGFP, AD-Sur-EGFP and AD-EGFP respectively at MOI of 10. a: AD-EGFP group b: ZD55-EGFP group c: AD-Sur-EGFP group d: ZD55-Sur-EGFP group. (A) RT-PCR showed significant reduction of Survivin mRNA in ZD55-Sur-EGFP and AD55-Sur-EGFP treated cells. (B) Survivin protein levels in above mentioned groups were consistent with mRNA expression by Westen blot, and XIAP protein expression was not affected. **P < 0.0001, *P < 0.05

RT-PCR was performed 48 h after infection at MOI of 10. Both ZD55-Sur-EGFP and AD-Sur-EGFP suppressed the expression of Survivin mRNA in SW480 and LoVo cells significantly, whereas ZD55-EGFP and Ad-EGFP showed little inhibition on Survivin mRNA. The Survivin protein expression analyzed by western blot was consistent with results from RT-PCR. The gels were analyzed by ImageMaster Total Lab software. Results showed ZD55-Sur-EGFP and AD-Sur-EGFP significantly down regulated the expression of Survivin protein but ZD55-EGFP and AD-EGFP had little effect on Survivin expression. Importantly, infection of neither ZD55-Sur-EGFP nor AD-Sur-EGFP affected the expression of another anti-apoptotic protein XIAP. (Fig 4)

### Inhibition on in vitro growth and viability

**Figure 5:**
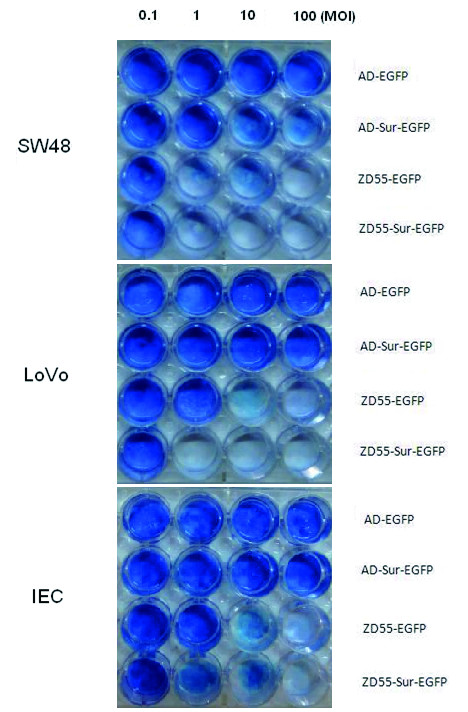
**The impact of oncolytic adenovirus mediated RNAi against Survivin on SW480, LoVo and IEC cells**. Cells were seeded in a 24-well plate at 1 × 10^5 ^cells per well. Then they were infected with different adenoviruses at different MOIs. At last, cells were stained with Coomassie brilliant blue.

To detect the specific cytopathic effect of ZD55-Sur-EGFP in tumor cells, SW480, LoVo, as well as IEC cells, were infected with various adenoviruses at indicated MOIs. As shown in Fig 5. Marked cytopathic effect was observed in both tumor cell lines infected with ZD55-Sur-EGFP compared with ZD55-EGFP, AD-Sur-EGFP and AD-EGFP infected cells even at low MOIs. And ZD55-Sur-EGFP caused limited cell death in normal cell line IEC.

**Figure 6:**
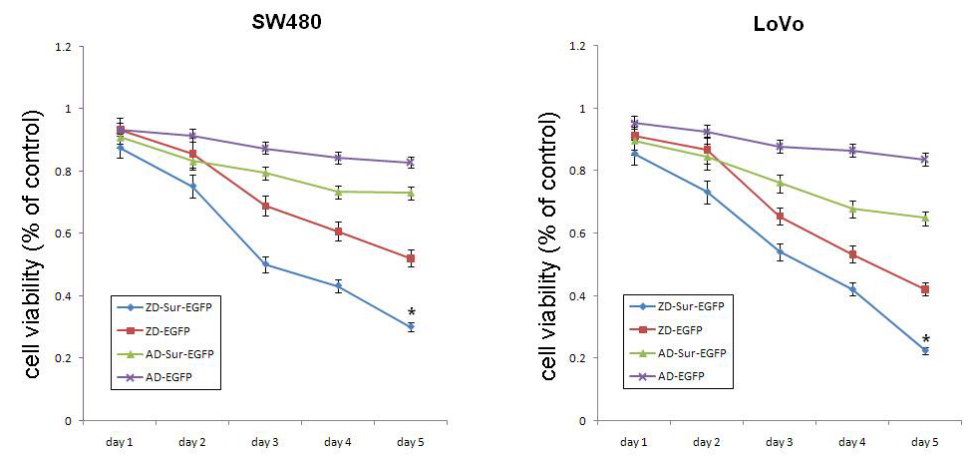
**Cells were transfected with ZD55-Sur-EGFP, ZD55-EGFP ADS-Sur-EGFP and AD-EGFP respectively at MOI of 5**. On 1 to 5 days post transfection, cells were subjected to MTT assay. This diagram shows the result of cell viability in each group. *P < 0.0001 vs other groups.

MTT cell viability assay demonstrated that SW480 and LoVo cells infected with ZD55-Sur-EGFP showed a significantly stronger CPE in a time-dependent manner. ZD55-Sur-EGFP could kill colorectal cancer cells more powerfully compared with other groups (Fig 6).

### Apoptosis induced by adenoviruses

**Figure 7:**
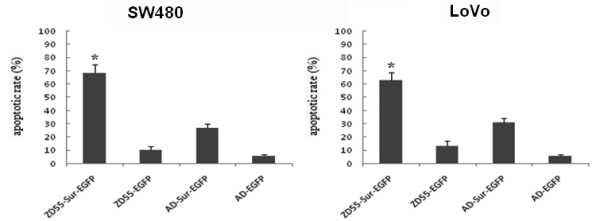
**Cell apoptosis was detected by flow cytometry**. The apoptotic rates of SW480 and LoVo cells infected with ZD55-Sur-EGFP were obviously higher (68.02% ± 6.88% and 63.79% ± 6.06%; P < 0.0001) than that of ZD55-EGFP (10.46% ± 2.31% and 13.38% ± 3.05%), AD-Sur-EGFP (27.57% ± 2.49% and 31.09% ± 2.68%) and AD-EGFP groups (6.14% ± 0.72% and 6.74% ± 0.47%).

As shown in Fig 7, the transfection of oncolytic adenoviruse with Survivin shRNA remarkably increased apoptotic populations in SW480 and LoVo cells by FCM analysis. The apoptotic rate in cancer cells transfected with ZD55-Sur-EGFP (68.02% and 63.79%) was of great statistic significance compared with ZD55-EGFP (10.46% and 13.38%), AD-Sur-EGFP (27.57% and 31.09%) and AD-EGFP (6.14 and 6.74%) groups

**Figure 8:**
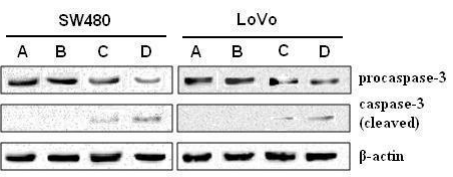
**Effect of adenoviruses on caspase-3 activity in SW480 and LoVo cells**. Western blot analysis was performed 48 h post infection. The activation of caspase-3 (demonstrated as increased expression of cleaved fragments of caspase-3) was more obvious in ZD455-Sur-EGFP group (D) than in AD-Sur-EGFP group (C), whereas AD-EGFP (A) and ZD55-EGFP (B) did not actvivate caspase-3.

To confirm the apoptosis was mediated by caspase activation, we next examined the caspase-3 activation by immunoblot analysis. In both SW480 and LoVo cells, the cleaved fragments of caspase-3 increased along with the decrease of procaspase-3 in ZD55-Sur-EGFP and AD-Sur-EGFP infected groups, and the activation of caspase-3 was more obvious in ZD55-Sur-EGFP group. Infections with ZD-EGFP and AD-EGFP did not affect the status of caspase-3 (Fig 8).

### Effects of AD-Sur-EGFP on in vivo xenograft tumor model

**Figure 9:**
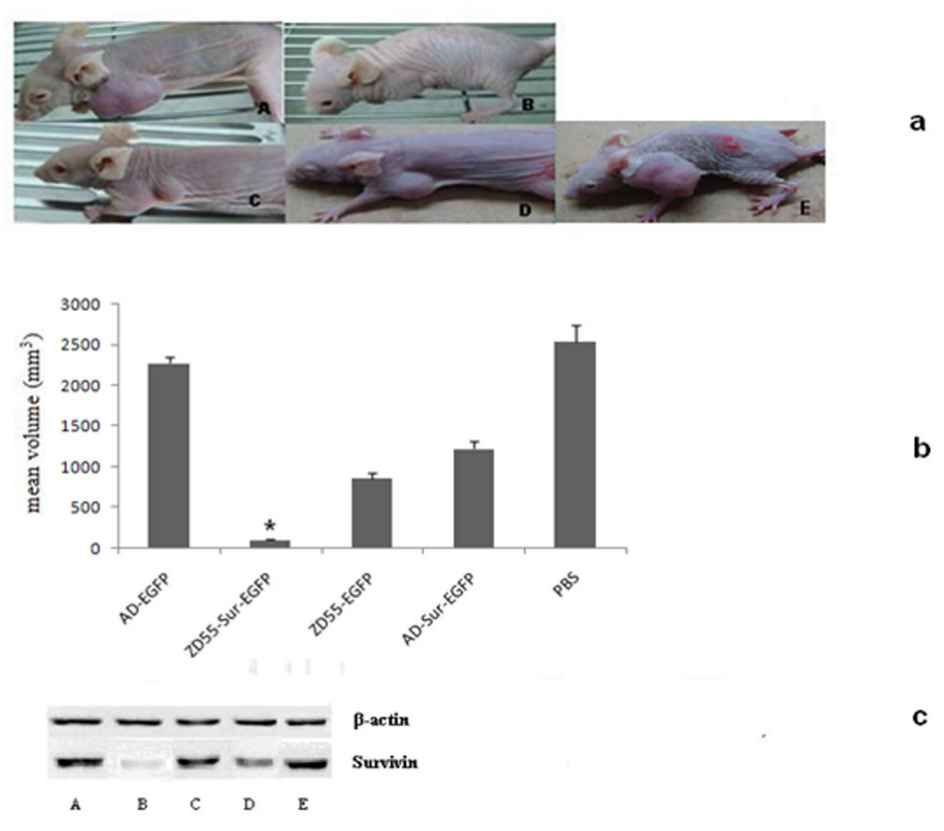
**Antitumor effects of oncolytic virus mediated Survivin RNAi in nude mice xenograft tumor model**. 4-week-old female BALBC/C nude mice were injected subcutaneously with SW480 cells and then with adenoviruses injected through the tail vein. After 60 days, the mice were sarcrificed and the tumors were harvested. A: AD-EGFP group B: ZD55-Sur-EGFP group C: ZD55-EGFP group D: AD-Sur-EGFP group E: PBS group (a) Representative tumor formations, 60 days after injection. (b) Tumor volume after 60 days of injection was quantitatively represented. Data were expressed as mean ± SD. *P < 0.01 vs other groups (c) Western blotting of proteins from xenograft tumors. The result was consistent with that on cell level.

To further investigate the antitumor effect of oncolytic adenovirus mediated Survivin knock down on the in vivo CRC tumor growth. SW480 cells suspended in serum free medium were subcutaneously implanted into nude mice and various adenoviruses were injected via tail vein. 60 days later the mice were sacrificed and tumors were resected. The PBS treated group outgrowth other groups (2536.44 mm^3 ^in volume). The mean volume of ZD55-Sur-EGFP group was 108.80 mm^3^, which was much smaller than the ZD55-EGFP group (863.56 mm^3^), AD-Sur-EGFP group (1224.97 mm^3^), AD-EGFP group (2278.21 mm^3^) and PBS treated group (Fig 9a,b).

### Western blot analysis of Survivin in xenograft tumors

To determine the effect of ZD55-Sur-EGFP on Survivin expression in vivo, we analyzed the Survivin protein in by western blot. As shown in Fig 9c, Survivin showed a marked reduction in ZD55-Sur-EGFP and AD-Sur-EGFP treated groups when compared with the PBS, AD-EGFP and ZD55-EGFP group. Furthermore, it is clearly that ZD55-Sur-EGFP suppressed Survivin expression more potent than AD-Sur-EGFP, and ZD55-EGFP, AD-EGFP and PBS had nearly no effect on Survivin expression.

## Discussion

Colorectal carcinoma is the most frequent alimentary system malignancy, which accounts for 40% of the estimated new cancer cases of the digestive tract [12]. Although the incidence of CRC in developed countries is slowly decreasing, it is increasing rapidly in developing countries. Treatments such as surgical operation, adjuvant chemotherapy and neo adjuvant chemotherapy have achieved great progress [13], but the reported survival rate of CRC within five years is not yet encouraging. The mortality of CRC is mainly due to metastasis to distant organs, especially to liver, which accounts for one-third of the metastatic colorectal cancers [14-18]. It is urgent to establish a more effective therapeutics for CRC.

RNA interference (RNAi) is a posttranscriptional gene silencing strategy first discovered in the nematode Caenorhabditis elegans [19]. Because of its high specifity and efficiency in down regulating gene expression, it has now become an excellent tool for exploring gene function. Many groups have worked on cancer gene silencing using RNAi in cell lines derived from different tissues, which lead to significant inhibition in cancer cell growth [20-24]. Also there are some in vivo studies using RNA interference strategies which achieve similar results [7,25]. But the transfection efficiencies of traditional RNAi strategies are relatively low. In order to facilitate the application of RNAi in cancer gene therapies, improved methods for efficient introduction of small interfering RNA (siRNA) into target cells are needed.

Oncolytic adenovirus as an anticancer agent is a potent treatment in various malignancies [26]. The best known oncolytic adenovirus named ONYX-015 is an E1B-55 kDa deficiency virus, which has shown promising results in head-and-neck cancer treatment combining with chemotherapy [27,28]. Another oncolytic adenovirus, H101, similar to ONYX-015, was recently approved by the Chinese government to be used in combination with radiotherapy for head-and-neck cancers too [29]. Many works have been done to increase the efficacy of oncolytic adenovirus virotherapy. Oliver and his colleagues constructed an oncolytic adenovirus expressing Herpes Simplex Virus-thymidine kinase which showed significant anti-neoplastic activity [30]. Another team from Taiwan used an E1B-deleted adenovirus driven by the squamous cell carcinoma cell antigen 2 promoter for uterine cervical cancer therapy [26]. Sagawa and his colleagues reported a successful inhibition of hepatocellular carcinoma by combining conditionally replicable adenovirus driven by α-fetoprotein enhancer/promoter (AFPep) with a replication-incompetent adenovirus carrying a p53 transgene also driven by AFPep [31]. But there is no report so far combining the oncolytic adenovirus with RNA interference in colorectal malignancy treatment.

ZD55 is a new E1B 55 kDa deleted adenovirus vector which replicates specifically in tumor cells and lyses them. Researchers had successfully armed different therapeutic genes with ZD55 and showed significant antitumor effects [32]. To improve the efficiency and potency of Survivin shRNA, we constructed ZD55-Sur-EGFP, an E1B 55 kDa deleted adenovirus carrying a Survivin targeted shRNA and a reporter gene.

In our study, we found the selectivity of ZD55-Sur-EGFP was much more obvious than that of AD-Sur-EGFP in colorectal cancer cell lines by reporter gene assay. We demonstrated that shRNA expressed from ZD55-Sur-EGFP significantly decreased Survivin expression of colorectal cancer cells as compared with AD-Sur-EGFP, but ZD55-EGFP and AD-EGFP had nearly no effect on Survivin expression. Moreover, the cytopathic effect of ZD55-Sur-EGFP on the tumor cell lines was more apparent than that of ZD55-EGFP, AD-Sur-EGFP and AD-EGFP. These results suggest the selectivity of ZD55 could amplify the copies of shRNA in tumor cells and allow the viral infection to adjacent tumor cells, which further enhanced the RNAi potency. Furthermore, the oncolytic effect and Survivin RNAi synergistically suppressed tumor cell growth, leading to significant cell death.

In our study, the data indicated ZD55-Sur-EGFP could induce much stronger apoptosis in both colorectal cancer cell lines than induced by ZD55-EGFP, AD-Sur-EGFP and AD-EGFP by activating caspases. Interestingly, we found infection of ZD55-EGFP had the potential to induce apoptosis, which was independent to Survivin regulation by RT-PCR and immunoblot analysis. A possible explanation is that some oncolytic virus structure proteins have an effect on the induction of tumor cell apoptosis and virus gene integration into the genome of cancer cells could lead to increased susceptibility to apoptosis [33].

In our present study, another interesting finding was that despite a remarkable induction of apoptosis as a consequence of the inhibition of Survivin after both infections of ZD55-Sur-EGFP and AD-Sur-EGFP, a significant decrease of cell viability was observed only after infection with ZD55-Sur-EGFP in MTT assay. This phenomenon could be explained by the characteristics of both virus vectors. AD-Sur-EGFP is a replication deficient adenovirus which cannot replicate in tumor cells, initiating a limited time of Survivin down regulation and cell apoptosis; on the contrary, ZD55-Sur-EGFP can selectively replicate in those cells, delivering Survivin shRNA and then lyses the cells. This explanation is further confirmed by MTT assay: during the first two days, the cell viabilities in AD-Sur-EGFP group was lower than in ZD55-EGFP group, but after 2 days, the cell viability in ZD55-EGFP group became lower than AD-Sur-EGFP group because of the replication of oncolytic virus.

Previous study has shown that adenovirus based RNAi against Survivin led to significant inhibition of Survivin expression and tumor growth in vivo [7]. Our xenograft tumor model demonstrated that ZD55-Sur-EGFP has a more potent antitumor activity than that of ZD55-EGFP, AD-Sur-EGFP and AD-EGFP. Besides the direct anticancer effect of the oncolytic virus itself, the much more efficient Survivin shRNA delivering, gene silencing and induction of apoptosis contribute greatly to the potent antitumor activity.

## Conclusion

In conclusion, the ZD55-Sur-EGFP has both the oncolytic ability and the capacity to deliver Survivin shRNA. This oncolytic adenovirus based Survivin RNA interference could efficiently reduce the cell growth, tumorigenicity and increase apoptosis of colorectal cancer cells, which offers a prospect of improvement in treatment of CRC, even a promising treatment for other human cancers.

## Competing interests

The authors declare that they have no competing interests.

## Authors' contributions

Wei Shen, Chun-Yi Wang and Zhong-Xue Fu designed the research; Wei Shen and Xue-Hu Wang participated in the cell research; Wei Shen carried out the animal study; all authors took part in result discussion and data analysis; Wei Shen wrote the paper. All authors read and approved the final manuscript.
